# Baseline Findings from Dual-Phase Amyloid PET Study in Newly Diagnosed Multiple Sclerosis: Exploring Its Potential as a Biomarker of Myelination and Neurodegeneration

**DOI:** 10.3390/jpm15110520

**Published:** 2025-11-01

**Authors:** José María Barrios-López, Eva María Triviño-Ibáñez, Adrián Piñeiro-Donis, Fermín Segovia-Román, María del Carmen Pérez García, Bartolomé Marín-Romero, Ana Romero Villarrubia, Virginia Guillén Martínez, José Pablo Martínez-Barbero, Raquel Piñar Morales, Francisco J. Barrero Hernández, Adolfo Mínguez-Castellanos, Manuel Gómez-Río

**Affiliations:** 1 Servicio de Neurología, Hospital Universitario Virgen de las Nieves, 18013 Granada, Spain; bartolome.marin.sspa@juntadeandalucia.es (B.M.-R.); ana.romero.villarrubia.sspa@juntadeandalucia.es (A.R.V.); virginia.guillen.sspa@juntadeandalucia.es (V.G.M.); adolfo.minguez.sspa@juntadeandalucia.es (A.M.-C.); 2 Servicio de Medicina Nuclear, Hospital Universitario Virgen de las Nieves, 18014 Granada, Spain; adrian.pinero.sspa@juntadeandalucia.es (A.P.-D.); manuel.gomez.rio.sspa@juntadeandalucia.es (M.G.-R.); 3 Instituto de Investigación Biosanitaria ibs. GRANADA, 18012 Granada, Spain; josep.martinez.sspa@juntadeandalucia.es (J.P.M.-B.); raquel.pinar.sspa@juntadeandalucia.es (R.P.M.); franciscoj.barrero.sspa@juntadeandalucia.es (F.J.B.H.); 4 Department of Signal Theory, Networking and Communications, University of Granada, 18016 Granada, Spain; fsegovia@ugr.es; 5 Servicio de Radiología, Hospital Universitario Virgen de las Nieves, 18014 Granada, Spain; mcarmen.perez.garcia.sspa@juntadeandalucia.es; 6 Servicio de Neurología, Hospital Universitario Clínico San Cecilio, 18016 Granada, Spain

**Keywords:** amyloid PET, multiple sclerosis, biomarker, myelination, neurodegeneration

## Abstract

**Background:** Amyloid positron emission tomography (PET) has been proposed as a tool to monitor myelination in multiple sclerosis (MS). We present baseline results from an ongoing prospective study, which is the first to include both early and standard phases of amyloid PET in patients with newly diagnosed MS. **Methods:** The prospective study includes patients with newly diagnosed MS (January 2023–February 2024). Clinical evaluation includes neurological disability (EDSS) and neuropsychological assessment. Brain MRI, early [^18^F]florbetaben (FBB) PET (eFBB; 0–5, 0–10 min post-injection), and standard FBB PET (sFBB; 90 min post-injection) were acquired. Normal-appearing white matter (NAWM) and damaged white matter (DWM) in MRI were segmented and co-registered with PET images. Results are presented as standardized uptake values (SUV), with the ratio using cerebellum as the reference region (SUVR) and the percentage of change between the DWM and NAWM. **Results:** Twenty patients were included (35.05 ± 10.72 years; 75% women). Both eFBB and sFBB acquisitions showed significantly lower SUVRmax and SUVRmean, and higher SUVRmin in the DWM compared to NAWM (*p* < 0.001) in all patients. SUV parameters in both DWM and NAWM from eFBB and sFBB PET correlated with the number of relapses and EDSS (r = −0.454 and r = −0.446, respectively; *p* < 0.05). Additionally, SUVR values in the DWM during eFBB correlated with cognitive impairment (SDMT; r = −0.516, *p* < 0.01), fatigue (MFIS-5; r = −0.450, *p* < 0.05), and quality of life (EQ-5D; r = −0.490, *p* < 0.05). **Conclusions:** Quantitative analysis of dual-phase FBB PET demonstrates differential uptake between DWM and NAWM, which is probably associated with demyelination and neurodegeneration. These preliminary findings suggest that amyloid PET may have predictive value for disease activity and progression, supporting its potential as a biomarker in MS. Follow-up data from this study are needed to support the baseline results.

## 1. Introduction

Multiple sclerosis (MS) is a chronic autoimmune disease of the central nervous system, characterized by inflammatory demyelinating and axonal injury, leading to secondary neurodegeneration [1]. MS typically begins between 20 and 40 years of age, is more frequent in women (female-to-male ratio, 3:1), and remains the leading cause of non-traumatic neurological disability in young adults in Europe and the United States [2]. The pathological hallmark includes focal white matter (WM) lesions with inflammation and demyelination. These lesions evolve into variable chronic states, including remyelination, unresolved inflammation, or persistent demyelination with axonal loss [1].

Relapsing–remitting MS (RRMS) is the most frequent initial phenotype (approximately 85%), characterized by clinical relapses with variable recovery, interspersed with periods of remission and disability progression independent of relapses [3]. Around 20% of patients later develop secondary progression, and approximately 15% of patients present with progressive disease from the onset. Progressive forms are associated with worse outcomes and greater disability [1,3].

Disability accumulation in MS is primarily driven by inflammatory activity and neurodegenerative progression [4]. Consequently, several tools have been developed for disease monitoring [5]. Clinically, the Expanded Disability Status Scale (EDSS) remains widely used. Brain magnetic resonance imaging (MRI) enables detection of demyelinating lesions and is essential for diagnosis and follow-up [1,5]. In recent years, neurofilament light chain (NfL) has emerged as a promising fluid biomarker associated with activity and progression [6]. However, both MRI-based advanced imaging markers and NfL present limitations, such as complex post-processing images requirements or biological variability influenced by individual factors [6,7,8]. Therefore, complementary biomarkers are needed to enhance the accuracy of risk stratification and personalized treatment decisions.

Amyloid positron emission tomography (PET) imaging has been proposed as a noninvasive technique for assessing myelin integrity in MS patients [9]. Amyloid tracers display selective affinity for aggregated β-sheet structures, such as the myelin basic protein, which is abundant in WM. When myelin is damaged, the loss of β-sheet conformation leads to reduced tracer binding [10]. Initial studies using amyloid PET in MS demonstrated lower uptake in damaged white matter (DWM) compared to normal-appearing white matter (NAWM) in both MS patients and healthy controls [11]. Subsequent investigations also detected reduced tracer uptake in MRI-defined NAWM, particularly in active RRMS and progressive phenotypes, when compared to stable RRMS patients [9,12,13]. Moreover, lower baseline amyloid uptake in the NAWM has been associated with a higher risk of cognitive decline and greater disability [14,15]. However, most existing studies are limited by cross-sectional designs, heterogeneous cohorts, not including newly diagnosed patients, and not analyzing the early PET phase [9,12,13,14,16].

We hypothesize that dual-phase amyloid PET may detect changes related to myelination and neurodegeneration in patients with MS. The primary aim is to evaluate dual-phase amyloid PET findings and their correlation with clinical markers of activity and progression. This is the first study to apply this approach in a homogeneous cohort of newly diagnosed RRMS patients.

## 2. Materials and Methods

### 2.1. Study Design, Population, and Sample Size Estimation

**Figure 1:**
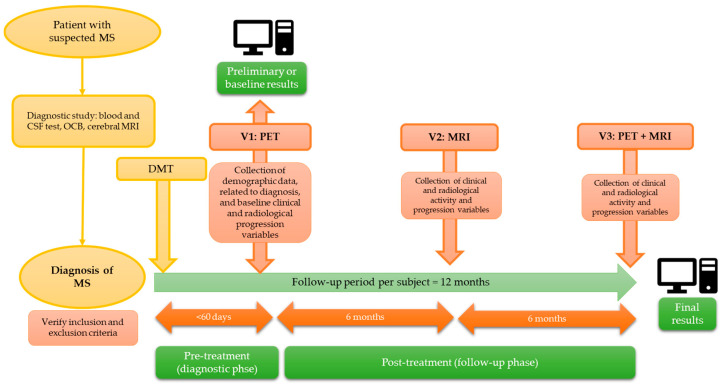
Baseline and follow-up period for the included participants. OCB (oligoclonal bands); MS (multiple sclerosis); CSF (cerebrospinal fluid); PET (positron emission tomography); MRI (magnetic resonance imaging); DMT (disease-modifying treatment); V1–3 (sequential visits: V1: baseline; V2: 6-month; V3: 12-month visit)**.**

We conducted a multicenter and prospective study in subjects with a recent diagnosis of RRMS, divided into two phases: a pre-treatment or diagnostic phase, in which the correlation between PET findings and baseline variables was analyzed, and a post-treatment or follow-up phase, involving a new follow-up PET scan at 12 months, in which the correlation between the baseline and follow-up PET findings and clinical/radiological markers of disease activity and progression was assessed. This article presents preliminary findings from the pre-treatment phase (Figure 1).

Due to the lack of previous similar studies, the sample size was determined by the number of patients diagnosed during recruitment, the number of participating centers, and the availability for neuroimaging (MRI and PET). All eligible patients who provided informed consent were included. For a paired *t*-test with a two-sided α of 0.05 and 80% power and based on previous studies, a minimal clinically relevant SUVR difference of 0.1 units and a standard deviation of 0.15 were assumed [9,15]. This required 18 paired observations, which were increased by 10% to account for potential dropouts, resulting in a final sample of 20 participants. This sample size also allows detection of a correlation of r ≥ 0.6 (two-sided, α = 0.05, 80% power, Fisher’s z-transformation).

A comprehensive and consecutive recruitment of patients was carried out after being diagnosed with RRMS (according to the 2017 revised McDonalds criteria), from January 2023 to March 2024, in two public hospitals in southern Spain (Hospital Universitario Clínico San Cecilio and Hospital Universitario Virgen de las Nieves in Granada) [17]. The inclusion and exclusion criteria for MS patients are listed in Supplementary Table S1.

### 2.2. Clinical and Cognitive Assessments

Demographic, clinical, radiological, and neuropsychological variables were collected from each subject during the pre-treatment (diagnostic) phase and subsequently during the post-treatment phase. The study variables are summarized in Supplementary Table S2.

Each subject underwent a clinical examination, including the assessment of physical disability through the Expanded Disability Status Scale (EDSS), the Timed 25-Foot Walk (T25FW) and the Nine-Hole Peg Test (9HPT) to assess walking speed and upper extremity dexterity, respectively [5]. Cognitive and neurobehavioral evaluation was assessed, including evaluation of information processing speed, verbal memory, and learning and visual memory through the Symbol Digit Modalities Test (SDMT), California Verbal Learning Test (CVLT), and the Brief Visuospatial Memory Test (BVMT), respectively. Additional tests included in the neuropsychological battery are summarized in Supplementary Table S2. Each patient also completed the Modified Fatigue Impact Scale (MFIS-5), the Beck Depression Inventory (BDI-II) to assess depressive symptoms, and EuroQoL (EQ-5D) for health-related quality of life [18].

Clinical assessments and scale administration were conducted by neurologists from the Demyelinating Diseases Units of the participating hospitals, who were blinded to data collection and analysis. Neuropsychological evaluation and the BICAMS battery were administered by a neuropsychologist.

### 2.3. Brain Magnetic Resonance Imaging

All patients underwent two MRI examinations: one at baseline and one during the follow-up. Both diagnostic and follow-up MRI studies were performed on a Philips Ingenia CX 3T system, with a total acquisition time of approximately 20 min. Sequence-specific MRI parameters are detailed in Supplementary Table S3. All the three-dimensional sequences were acquired with an isotropic voxel size. The images obtained were evaluated by a neuroradiologist with 3 years of experience, considering target lesions with enhancement or with a high signal in T2/FLAIR and a diameter above 3 mm, with a maximum of 5 lesions of each type per patient.

### 2.4. Positron Emission Tomography with [^18^F]Florbetaben

Amyloid PET images were acquired in a Siemens Biograph Vision 600 Positron Emission Tomography/Computed Tomography (PET/CT) digital scanner. The tracer used, [^18^F]florbetaben (FBB) (Neuraceq; Life Molecular Imaging, Berlín, Alemania), was administered intravenously at a mean dose of 300 MBq. Amyloid PET was acquired with a dual-point protocol including two acquisitions: early FBB PET (eFBB; 0–5 and 0–10 min post—injection), and standard acquisition FBB PET (sFBB; 90 min post—injection). Specific details about the imaging protocol are given in Supplementary Table S4.

### 2.5. Neuroimaging Pre-Processing and Analysis

**Figure 2:**
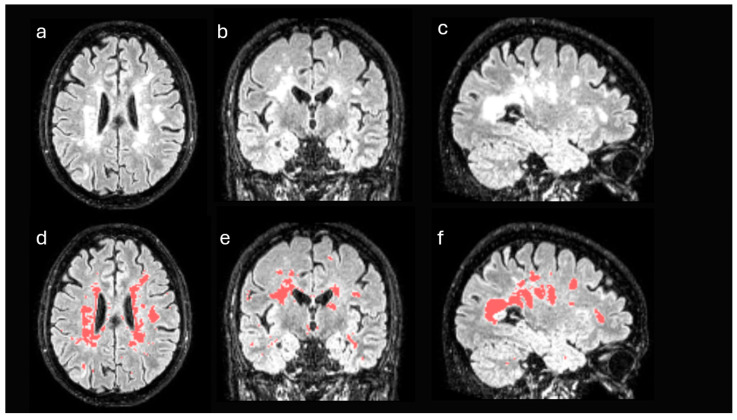
Damaged white matter segmentation on FLAIR-MRI. Axial (**a**), coronal (**b**), and sagittal (**c**) views. Lesions were identified using a semi-automated thresholding method. Damaged areas are highlighted in red in (**d**–**f**).

Image processing was performed using a structured workflow, which included lesion segmentation, spatial registration of the data and calculation of standardized uptake values (SUVs) in different brain regions (Figure 2).

First, structural MRI FLAIR images of each patient were processed in order to identify disease-specific lesions. These images were registered to the standard Montreal Neurological Institute (MNI) space and segmented using the unified segmentation algorithm implemented in Statistical Parametric Mapping (SPM), version 12 [19]. After the process, the gray matter and white matter of each patient were selected in the MNI space, discarding the remaining tissues. From these data and using a semi-supervised thresholding procedure, a mask with the lesions of each patient was generated (Figure 2). Using these masks, the lesion volume of each patient was obtained, both globally in the whole brain and in isolation in WM. Likewise, the masks were used to estimate the volume of the non-damaged white matter of each patient, that is, the white matter excluding the areas affected by the disease.

On the other hand, amyloid PET images were spatially registered to the MNI space to ensure anatomical correspondence with the processed MRI images. The registration was performed using rigid alignment transformations and nonlinear alignment transformations, optimized to preserve the anatomical relationship between the modalities [20].

Once the spatial registration was completed, we proceeded to the quantitative analysis of the PET images by calculating SUV in different regions of interest (ROIs). ROIs were defined based on the masks obtained in the previous steps and comprised the following: DWM, NAWM, non-damaged gray matter (GM), total GM, and cerebellum. The gray matter and WM masks were derived from two complementary sources: the segmentation of FLAIR MRI images and the segmentation of structural T1 MRI images from the same patients, allowing the comparison of the robustness of both approaches.

The following SUVs were calculated for both the DWM and the NAWM:

- SUVmax (maximum standardized uptake value): The highest SUV value within a defined region of interest (ROI). It reflects the point of greatest radiotracer uptake and is commonly used as an indicator of peak metabolic activity.
- SUVmean (mean standardized uptake value): The mean SUV within the ROI represents the average value of all voxel SUV measurements. It provides a robust measurement that is less influenced by outliers compared to SUVmax.
- SUVmin (minimum standardized uptake value): The lowest SUV value within the ROI. While less frequently used on its own, it can be helpful in assessing areas of low tracer uptake or evaluating lesion heterogeneity.
- SUVR (SUV relative to the cerebellum): Ratio using the cerebellum as the reference region.
- % of change SUV: Percentage of change between the DWM and NAWM calculated, according to previous studies, as follows: the DWM uptake minus NAWM uptake, divided by the NAWM uptake and multiplied by 100 [9].

The delineation of the cerebellum was performed from the Automated Anatomical Labeling (AAL) anatomical atlas, registered to the same MNI space [21]. All masks were adjusted to the resolution and dimensions of the PET images before the calculation of SUVs, to avoid interpolation errors or incorrect overlapping between modalities.

All MRI and PET images were analyzed by raters blinded to clinical and neuropsychological data.

### 2.6. Standard Protocol Approvals, Registrations, and Patient Consents

This study was performed in accordance with Good Clinical Practice and the Helsinki Declaration and was approved by the Biomedical Research Ethics Committee of the Province of Granada, under the code AMPET-MS22 (107-N-22), on 29 July 2022. Informed consent was obtained from all study participants after reviewing the inclusion and exclusion criteria.

### 2.7. Statistical Analysis

Descriptive data are shown as means ± standard deviations or as frequencies and percentages. To compare the SUV parameters in different regions within the same patient, paired Student’s *T*-test was used after confirming normal distribution with the Kolmogorov–Smirnov test; for non-normally distributed variables, the Wilcoxon signed-rank test was applied. A repeated-measures ANOVA was performed to evaluate the evolution of SUV parameters from the early phases (5 and 10 min) to the standard phase. The assumption of sphericity was tested using Mauchly’s test and found to be violated; therefore, the Greenhouse–Geisser correction was applied. Correlations between neuroimaging measures and clinical and neuropsychological tests were calculated using Spearman’s coefficient. SPSS 29.0 (IBM SPSS, Armonk, NY, USA) and R version 4.2.0 (R Foundation for Statistical Computing, Vienna, Austria) were used for data analyses. Statistical significance was set at *p* < 0.05.

## 3. Results

### 3.1. Baseline Clinical and Neuroimaging Characteristics

Between January 2023 and February 2024, 20 patients were included (75% female), with a mean age at diagnosis of 35.05 ± 10.72 years. The mean number of relapses and EDSS score at diagnosis were 1.95 ± 1.15 and 1.90 ± 1.09 (median 2), respectively (Table 1). Additional baseline demographic, clinical, and neuropsychological variables are summarized in Supplementary Table S5.

In the neuroimaging analysis, patients showed a mean (±standard deviation) MRI volume of 13.94 ± 14.98 cm^3^ in the DWM, 540.98 ± 38.59 cm^3^ in the NAWM, and 968.63 ± 72.70 cm^3^ in the GM. Regarding the DWM lesion load, two patients had a low lesion burden (≤9 lesions on T2/FLAIR), eight patients had a high non-confluent lesion burden (>9 lesions on T2/FLAIR), and ten patients presented with a high confluent lesion burden (>9 lesions on T2/FLAIR with extensive, confluent and/or uncountable lesions). Co-registration of MRI and FBB PET yielded a total of 54 variables derived from the analysis of SUV parameters in the DWM and NAWM across both phases of the FBB PET. These are summarized in Supplementary Table S6.

### 3.2. Comparison of Tracer Uptake Intensity in DWM Versus NAWM

**Figure 3:**
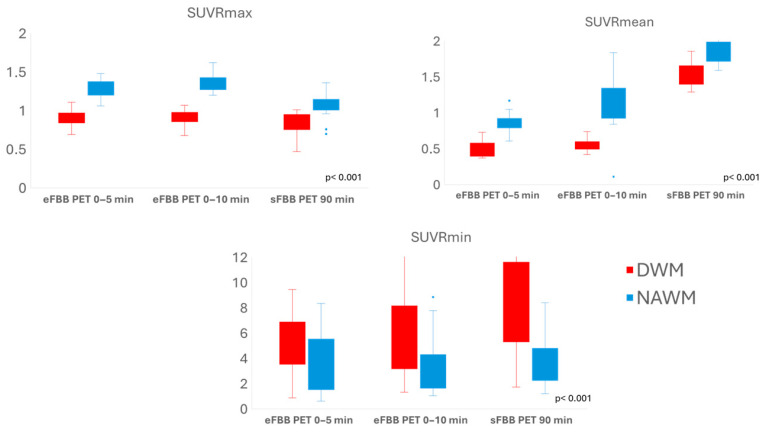
Box plot illustrating the comparison of SUVR (see definitions in “Neuroimaging Pre-processing and Analysis” subsection) in the damaged (DWM) versus normal-appearing white matter (NAWM) across early (eFBB) and standard (sFBB) phases of [^18^F]florbetaben PET. Mean SUVRmax and SUVRmean values in the DWM were significantly lower than those in the NAWM in both eFBB and sFBB. In contrast, mean SUVRmin values in the DWM were significantly higher than those in the NAWM.

Both eFBB and sFBB PET acquisition showed significantly lower mean SUVmax and SUVmean in the DWM compared to the NAWM. However, the SUVmin in the DWM were significantly higher than those in the NAWM. Similarly, the DWM showed significantly lower SUVRmax and SUVRmean (cerebellum-referenced SUVmax and SUVmean, respectively) compared to the NAWM, while SUVRmin were higher in the DWM than in the NAWM (Figure 3; Supplementary Table S7).

### 3.3. Comparison of SUVR Values in DWM and NAWM Between Early and Late FBB PET Phases

A repeated-measures analysis revealed that SUVR values in both the DWM and NAWM, as well as the % of change between the DWM and NAWM (% of change in SUV), changed significantly from eFBB to sFBB acquisition. The SUVRmax values increased during eFBB and then decreased in the sFBB, while SUVRmean and SUVRmin showed a continuous upward trend from eFBB to sFBB, predominantly in DAWM (Table S8; Figure S1, Supplementary Material).

### 3.4. Correlations Between FBB PET Quantitative Parameters and Clinical/Neuropsychological Scales

**Figure 4:**
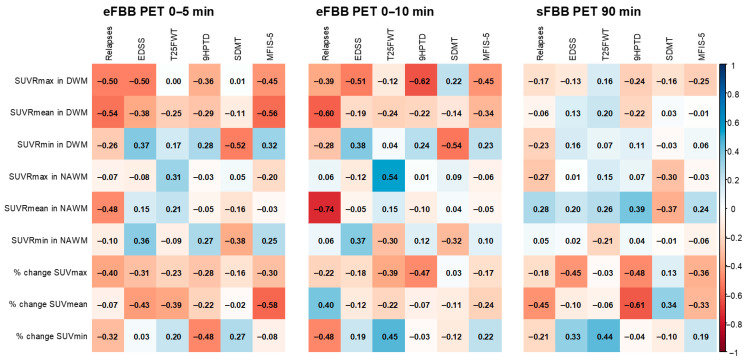
Correlogram visualizing correlation between SUV parameters calculated in eFBB and sFBB phases of PET and clinical variables related to disease activity and progression. Color shades of the cells are proportional to the correlation coefficients. Positive correlations are shown in blue whereas negative correlations in red (dark blue and dark red with the strongest correlation, respectively).

Correlations with clinical variables related to disease activity, progression, and neuropsychological function are summarized in Figure 4 and Supplementary Table S9. The number of relapses within one year showed a significant negative correlation with SUVRmean values in both the DWM and NAWM in the eFBB (r = −0.541 and r = −0.596, respectively; *p* < 0.05), and with the % of change in SUVmean in the sFBB phase (r = −0.454, *p* < 0.05).

EDSS scores were significantly and negatively correlated with SUVRmax in the DWM in the eFBB phases (r = −0.501 and r = −0.513, *p* < 0.05) and with the % of change in SUVmax in the DWM in the sFBB phase (r = −0.446, *p* = 0.049) (Supplementary Figure S2). Manual dexterity, as measured by the 9HPTD, was significantly and inversely correlated with the % of change in SUVmax in both eFBB and sFBB (r = −0.472 and r = −0.477, *p* < 0.05, respectively). Gait speed, assessed using the T25FW, was positively correlated with the % of change in SUVmin in both eFBB and sFBB (r = 0.453 and r = 0.440, *p* < 0.05, respectively).

Regarding neuropsychological evaluation, a significant negative correlation was found between information processing speed (SDMT) and SUVRmin in the DWM in eFBB phases (r = −0.516 and r = −0.539, *p* < 0.05, respectively). Verbal memory and learning, measured by the CVLT, also showed significant negative correlations with SUVRmin in both the DWM and NAWM in eFBB (r = −0.564 and r = −0.649, respectively; *p* < 0.05). Symptoms of depression (BDI-II) were significantly and positively correlated with SUVRmin in the DWM in eFBB (r = 0.570 and r = 0.444, *p* < 0.05, respectively).

Fatigue, as assessed using the MFIS-5, showed a significant negative correlation with SUVRmax in the DWM in the eFBB phases (r = −0.450, *p* < 0.05 at both 5 and 10 min). Finally, quality of life (EQ-5D) was negatively correlated with SUVRmin in the DWM in eFBB phases (r = −0.468 and r = −0.490, *p* < 0.05).

## 4. Discussion

Amyloid PET has been proposed to monitor myelination changes in patients with MS, showing reduced SUVR in the DWM compared to NAWM on MRI in MS patients [11]. Greater NAWM demyelination has been observed in more active patients or progressive forms, and lower baseline SUVR in the NAWM has been associated with increased risk of disability progression (EDSS) and cognitive impairment (SDMT) [9,12,13,14,15].

We present preliminary findings from our study, based on co-registration of FBB PET and MRI, and their association with clinical variables at diagnosis. Our work is the first to include a homogeneous cohort of newly diagnosed RRMS patients [9,12,13,14,15,16]. This is particularly relevant, as acute inflammatory activity is the highest during the early stages of MS, allowing us to analyze baseline PET findings and identify patients at greater risk of long-term disease activity and progression [22]. Finally, this is the first work to physiologically interpret the eFBB and the SUV parameters, exploring their potential utility in detecting neurodegeneration- and inflammation-related changes [23].

### 4.1. Myelination and Neurodegeneration

At diagnosis, RRMS patients showed lower SUVRmax and SUVRmean values in the DWM compared to the NAWM. These findings can be explained by several factors: the affinity of amyloid tracers for β-sheet-structured protein such as myelin basic protein, the greater degree of demyelination observed in the DWM in pathological studies, and similar results from previous amyloid PET studies in MS, which demonstrated lower tracer uptake in the DWM areas compared to NAWM in both MS patients and healthy controls [9,11,24]. Altogether, these results reinforce the potential use of FBB PET to detect dynamic in vivo changes in myelination.

On the other hand, we observed that SUVRmin values were higher in the DWM than in NAWM. This finding is novel, as most previous studies have focused on SUVRmax or the % of change between the DWM and NAWM, with lower values associated with greater demyelination [9,14,15]. Several explanations may account for this result. First, neuropathological studies support that soluble Aβ oligomers have been detected in the brain tissue of MS patients using oligomer-specific antibodies [25]. A recent study conducted in a mouse model of MS observed Aβ oligomer deposits in the white matter, colocalizing with activated microglia, increasing during the most inflammatory phase, and stabilizing during the chronic phase [26]. Moreover, amyloid precursor protein is expressed in reactive astrocytes, microglia, T cells, and damaged axons within actively demyelinating MS plaques and in more chronic lesions [23,27]. Reduced CSF Aβ1-42 levels have been associated with a higher risk of disability progression and with increased pro-inflammatory cytokines in RRMS patients, supporting a link between amyloid metabolism, inflammation, and neurodegeneration [28,29]. Taken together, these findings suggest that SUVRmin may capture amyloid-related accumulation associated with neurodegeneration and/or inflammation [23]. This hypothesis is consistent with our findings, as a greater degree of neurodegeneration would be expected in the DWM compared to NAWM. Thus, SUVRmin could provide complementary information to SUVRmax, serving as a potential marker of underlying neurodegeneration with or without concurrent inflammation. Nevertheless, these findings should be interpreted with caution in the absence of comparable studies and re-evaluated with the results of our work.

### 4.2. Early Phase of Amyloid PET

This is the first study to include the eFBB in patients with MS. Based on our findings, we propose an interpretation grounded in the temporal evolution of SUVR values from the eFBB to the sFBB (Table S8; Figure S1, Supplementary Material). SUVRmax tends to decrease from eFBB to sFBB, which would be the expected evolution considering the tracer’s pharmacokinetics, with lower SUVR in sFBB likely reflecting greater demyelination [9,15]

In contrast, both SUVRmean and SUVRmin exhibit an increasing trend from eFBB to sFBB. These findings are more challenging to interpret, as no previous studies have assessed both eFBB and sFBB of amyloid PET in MS patients. Initially our hypothesis was that eFBB uptake could be related to the inflammation activity (increased perfusion and edema), but our results are in consonance with the emerging evidence, suggesting that eFBB may reflect cerebral hypometabolism associated with neurodegeneration and synaptic dysfunction, in a manner similar to [^18^F]FDG PET [30]. In a study including 103 patients with cognitive impairment due to Alzheimer’s disease and 33 healthy controls, eFBB showed a strong correlation with [^18^F]FDG PET for the detection of regional hypometabolism in dementia patients [31]. Therefore, the lower SUVRmean and SUVRmin in the eFBB in our study suggest that eFBB could be a useful approach for detecting cerebral hypometabolism associated with neurodegeneration in MS patients. However, these findings are limited by the absence of previous studies including eFBB in MS patients, as well as by potential confounding factors that may influence the results, such as perfusion abnormalities, edema, and tracer kinetics.

### 4.3. Correlation with Clinical Variables Related to Disease Activity and Progression

A higher number of relapses in the year prior to diagnosis was associated with greater demyelination and neurodegeneration in the DWM and NAWM, reflected by lower % of change in SUVmean in sFBB and SUVRmean in eFBB. These findings are consistent with those reported by Pietroboni et al., who identified greater NAWM demyelination in patients with higher acute inflammatory activity (defined by a higher relapse rate and the presence of new MRI lesions) [13].

Higher disability scores, as measured by the EDSS, were also associated with increased demyelination and neurodegeneration in the DWM, reflected by lower % of change in SUVmax in sFBB and lower SUVRmax in eFBB. These results support previous studies linking greater demyelination to higher EDSS scores and in progressive forms of MS compared to RRMS [9,12,15]. Moreover, our data show that reduced manual dexterity (9HPTD) was associated with increased demyelination and neurodegeneration (lower % of change in SUVmax in both eFBB and sFBB), while impaired gait function (T25FW) was linked to neurodegeneration (lower % of change in SUVmin in both eFBB and sFBB). These findings support the association between FBB PET-detected demyelination and neurodegeneration with higher scores on disability progression scales.

In terms of cognition, previous studies have reported an increased risk of cognitive impairment—particularly in visuospatial function and working memory—in patients with lower white matter tracer uptake [15]. In our study, we also found that reduced processing speed (SDMT), verbal memory, and learning (CVLT) were associated with increased neurodegeneration in the DWM and NAWM, reflected by higher SUVRmin in eFBB. Additionally, greater depressive symptoms, as measured by the BDI-II, were correlated with increased neurodegeneration in both the DWM and NAWM (higher SUVRmin in eFBB). Finally, fatigue scores using the MFIS-5 were higher in patients with more demyelination and neurodegeneration in the DWM (lower SUVRmax in eFBB).

### 4.4. Study Strengths

The strengths of our study, not present in previous reports, include a homogeneous cohort of newly diagnosed RRMS patients, the potential for long-term longitudinal follow-up, the inclusion of eFBB acquisitions, and the analysis of distinct SUVR parameters across acquisitions.

### 4.5. Study Limitations

Study limitations include the interpretation of eFBB findings and the various SUV parameters, given the absence of prior studies including and analyzing them. Nevertheless, prior research supports the use of eFBB for detecting cerebral hypometabolism associated with neurodegeneration in other neurological diseases [30]. We did not include a control group, although this was not the primary objective of our study, as previous work has already demonstrated differences compared to healthy controls [12,14,15]. The sample size is limited, but comparable to other studies (*n* < 30), and this pilot study aims to evaluate the clinical utility of FBB PET in newly diagnosed MS patients, with the future goal of expanding the patient cohort. Our study only includes RRMS patients; therefore, the baseline results cannot be extrapolated to patients with progressive forms. However, longitudinal follow-up will identify those who evolve to progressive forms and assess whether FBB PET can predict the risk of progression.

## 5. Conclusions

MS remains the leading cause of non-traumatic neurological disability in young adults, and there is a growing need for complementary biomarkers to improve risk stratification and guide personalized therapeutic strategies. This pilot study is the first to evaluate dual-phase FBB PET in newly diagnosed MS patients, including early-phase analysis and distinct SUV parameters.

Preliminary findings suggest that quantitative analysis of dual-phase FBB PET can detect differential uptake between the DWM and NAWM in newly diagnosed MS patients, likely reflecting demyelination and neurodegeneration. The correlation with clinical markers of disease activity, progression, cognitive dysfunction, and fatigue supports the potential role of FBB PET as a biomarker in MS. Final results of our ongoing study, together with further research, are needed to confirm the prognostic value and clinical applicability of this approach for stratifying patients at higher risk of disease activity and progression, with important prognostic and therapeutic implications.

## Figures and Tables

**Table 1:** Clinical–demographic characteristics and neuropsychological testing of the study cohort.

Characteristic (*n* = 20)	Mean (SD) or *n* (%)
MS onset age (y)	35.05 (10.72)
Sex	
Male	5 (25)
Female	15 (75)
Initial clinical presentation	
Optic neuritis	6 (30)
Myelitis	7 (35)
Hemispheric syndrome	2 (10)
Brainstem syndrome	5 (25)
N° of relapses	1.95 (1.15)
EDSS score	1.90 (1.09)
Other progression disease scales	
T25FW (s)	5.62 (1.19)
9HPT-D (s)	23.66 (5.06)
9HPT-ND (seconds)	24.07 (3.49)
Fatigue: MFIS-5 score	8.95 (6.37)
Cognitive functions	
SDMT (z-score)	−1.13 (0.96)
CVLT-II (z-score)	−1.35 (1.18)
BVMT (z-score)	−0.68 (1.51)
Beck Depression Inventory-II (points)	16.55 (12.97)
Quality of life: EQ-5D (points)	68.75 (22.35)

SD: standard deviation; EDSS: Expanded Disability Status Scale; T25FW: Timed 25-foot Walk, 9HPT-D: Nine-hole Peg Dominant Side, 9HPT-ND: Nine-hole Peg Non-dominant Side Test, MFIS: Modified Fatigue Impact Scale; SDMT: Symbol Digit Modalities Test; CVLT-II: California Learning Verbal Test—Second Edition; BVMT-R: Brief Visuospatial Memory Test—Revised; PVF: phonemic verbal fluency; SVF: semantic verbal fluency; EQ-5D: index and visual analogy scale.

## Data Availability

All collected data were anonymized and stored in institutional databases and repositories to ensure the confidentiality and privacy of study participants, in accordance with the current legislation on personal data protection, specifically the General Data Protection Regulation (EU) 2016/679 (GDPR). Due to ethical and legal considerations regarding patient confidentiality and compliance with the General Data Protection Regulation (EU) 2016/679, the anonymized datasets generated and/or analyzed during the current study are not publicly available. For any clarification, the datasets are available from the corresponding author on reasonable request.

## Supplementary Materials

The following supporting information can be downloaded at: https://www.mdpi.com/article/10.3390/jpm15110520/s1, Figure S1: Multiple Line Chart showing comparative analysis of Standardized Uptake Value ratios (SUVR) between the early (eFBB) and standard (sFBB) phases of [^18^F]Florbetaben PET in both damage white matter (DWM) and normal-appearing white matter (NAWM); Figure S2: Scatter plot showing the correlation between EDSS score and the percentage of change in SUVmax calculated from standard-phase [^18^F]Florbetaben (sFBB) PET; Table S1: Inclusion and exclusion criteria for MS patients; Table S2: Study variables; Table S3: Sequence-specific MRI parameters; Table S4: Protocol details to acquire FBB PET data according with international guidelines; Table S5: Demographic characteristics, clinical assessment, and neuropsychological testing of the study cohort; Table S6: Structural (MRI) and functional (amyloid PET) neuroimaging characteristics of the study cohort; Table S7: Comparative analysis of Standardized Uptake Value (SUV) in damage white matter (DMG) vs. normally-appearing white matter (NAWM) in the early (eFBB) y standard (sFBB) phases of [^18^F] Florbetaben (FBB) PET; Table S8: Comparative analysis of Standardized Uptake Value ratios (SUVR) between the early (eFBB) and standard (sFBB) phases of [^18^F]Florbetaben PET in both damage white matter (DWM) and normal-appearing white matter (NAWM).; Table S9: Correlation between quantitative parameters from the early (eFBB) and standard (sFBB) phases of [^18^F]Florbetaben PET and clinical variables related to disease activity and progression.

## Abbreviations

The following abbreviations are used in this manuscript:

Aβ	Amyloid-β1-42 oligomers	NAWM	Normal-appearing white matter
BDI-II	Beck Depression Inventory-II	PET	Positron emission tomography
CSF	Cerebrospinal fluid	RRMS	Relapsing–remitting multiple sclerosis
CVLT	California Verbal Learning Test	sFBB	Standard acquisition [^18^F]florbetaben
DWM	Damaged white matter	SMDT	Symbol Digit Modalities Test
EDSS	Expanded Disability Status Scale	SUV	Standardized uptake value
eFBB	Early acquisition [^18^F]florbetaben	SUVmax	Maximum standardized uptake value
EQ-5D	EuroQoL-5D	SUVmean	Mean standardized uptake value
FBB	[^18^F]florbetaben	SUVmin	Minimum standardized uptake value
GM	Gray matter	SUVR	Standardized uptake value relative to the cerebellum
MFIS-5	Modified Fatigue Impact Scale 5-item Version	T25FW	The Timed 25-Foot Walk
MRI	Magnetic resonance imaging	9HPT	The Nine-Hole Peg Test
MS	Multiple sclerosis	% of change SUV	Percentage of change between SUV of DWM and NAWM
