# Supplementary material for: Baseline Findings from Dual-Phase Amyloid PET Study in Newly Diagnosed Multiple Sclerosis: Exploring Its Potential as a Biomarker of Myelination and Neurodegeneration
Source: J Pers Med. 2025 Nov 1;15(11):520. doi: 10.3390/jpm15110520 (PMC12653326; doi:10.3390/jpm15110520)
Supplement: Supplementary file 1 [file jpm-15-00520-s001.zip › jpm-3803386-supplementary.pdf]

SUPPLEMENTARY MATERIALS

Supplementary table S1. Inclusion and exclusion criteria for MS patients

**INCLUSION CRITERIA**
Age ≥ 18 years
Recent diagnosis of relapsing-remitting multiple sclerosis (based on the 2017 revised McDonald criteria) [1]
Ability to provide informed consent and comply with study protocol
**EXCLUSIÓN CRITERIA**
Age ≥ 60 years or pregnancy
More than 60 days since brain magnetic resonance imaging acquisition
More than 60 days since initiation of disease-modifying therapy
Clinically or radiologically isolated syndrome, or progressive forms of multiple sclerosis
Coexisting autoimmune overlap syndromes (e.g., Sjögren's syndrome)
History of other neurological or psychiatric disorders, or brain lesions unrelated to multiple sclerosis

1. Thompson AJ, Banwell BL, Barkhof F, Carroll WM, Coetzee T, Comi G, et al. Diagnosis of multiple sclerosis: 2017 revisions of the McDonald criteria. Lancet Neurol. 2018;17(2):162–73.

Supplementary table S2. Study variables

**OUTCOME VARIABLE:** Responder status (yes/no): Patients were classified as responders if they met the NEDA-3 criteria during follow-up.	
PREDICTOR VARIABLES	
Primary predictor variables:	Secondary predictor variables:
**Amyloid tracer uptake intensity in DWM and NAWM regions segmented from MRI was measured using the standardized uptake value (SUV; mean ± SD),** according to standard methodology available at the institution [1]. The following parameters were obtained:<br>• SUVmax (Maximum Standardized Uptake Value): The highest SUV value within a defined region of interest (ROI).<br>• SUVmean (Mean Standardized Uptake Value): The mean of SUV within the ROI, representing the average value of all voxel SUV measurements.<br>• SUVmin (Minimum Standardized Uptake Value): The lowest SUV value within the ROI. SUVR (SUV relative to the cerebellum): ratio using cerebellum as the reference region.<br>• % of change SUV: Percentage of change between DWM and NAWM calculated, according to previous studies, as follows: DWM uptake minus NAWM uptake, divided by NAWM uptake and multiplied by 100.	• **Demographic and Baseline Characteristics**: Date of birth/age; sex; smoking status; employment status; Charlson Comorbidity Index (0–33 points).<br>• **Clinical Variables Related to MS Activity and Progression**: Number of relapses prior to diagnosis; initial clinical syndrome; presence of oligoclonal bands in cerebrospinal fluid (yes/no); Expanded Disability Status Scale (EDSS; score 0–10); Timed 25-Foot Walk Test (T25FWT; seconds); 9-Hole Peg Test – Dominant Hand (9HPT-D; seconds) and Non-Dominant Hand (9HPT-ND; seconds); Modified Fatigue Impact Scale 5-item version (MFIS-5; score 0–20); EuroQol-5D (EQ-5D; score in points).<br>• **MRI variables**: Number of lesions on T2-weighted and gadolinium-enhancing sequences; volumes of damaged white matter (DWM), normal-appearing white matter (NAWM), and gray matter (GM) (in cm^3); DWM lesion burden categorized as follows: Low lesion burden (≤9 lesions on T2/FLAIR), High non-confluent lesion burden (>9 non-confluent lesions on T2/FLAIR), High confluent lesion burden (>9 lesions on T2/FLAIR with extensive, confluent, and/or uncountable lesions); Other central nervous system involvement: spinal cord, brainstem, and/or cerebellum<br>• **Neuropsychological Variables** (scores were corrected for age, gender, and education):<br>◦ Cognitive tests included in the BICAMS battery: SDMT, CVLT-II and BVMT.<br>◦ Additional tests from the neuropsychological battery: Stroop Color and Word Test, Digit Span Forward and Backward, Semantic Verbal Fluency Test, Phonological Verbal Fluency Test, and Beck Depression Inventory-II (BDI-II).<br>• **Clinical progression variables:** Number of relapses during follow-up, EDSS (scale 0–10), T25FW (seconds), 9HPT-D and 9HPT-ND (seconds)<br>• **Radiological progression variables (MRI):** number of new lesions on T2/FLAIR sequences and number of new gadolinium-enhancing lesions.<br>• **Dates collected**: Date of diagnosis, and dates of PET and MRI acquisition.<br>• **Corticosteroid treatment**: Previous or subsequent corticosteroid use prior to baseline PET (yes/no).

1. Segovia F, Gómez-Río M, Sánchez-Vañó R, Górriz JM, Ramírez J, Triviño-Ibáñez E, et al. Usefulness of dual-point amyloid PET scans in appropriate use criteria: A multicenter study. Journal of Alzheimer's Disease. 2018;65(3):765–79
NEDA-3 (No Evidence of Disease Activity-3); DWM: damage white matter; NAWM: normally-appearing white matter; SD: standard deviation; BICAMS: Brief International Cognitive Assessment for MS; SDMT: Symbol Digit Modalities Test; CVLT-II: California Learning Verbal Test – Second Edition; BVMT-R: Brief Visuospatial Memory Test-Revised

Supplementary table S3. Sequence-specific MRI parameters.

DEVICE: Philips Ingenia CX 3T system	
SEQUENCIES:	
2D Diffusion Weighted Imaging (DWI, b1000)	
Plane	axial
Repetition time (ms)	3642
echo time (ms)	79
slice thickness (mm)	4
spacing	0
matrix	256 x 256
field of view (cm)	23 x 23
3D T1-weighted Turbo Spin Echo (TSE) without contrast	
Plane	axial
Repetition time (ms)	7.9
echo time (ms)	3.5
slice thickness (mm)	1
spacing	0
matrix	256 x 256
field of view (cm)	25 x 20
3D T2-weighted TSE	
Plane	axial
Repetition time (ms)	2500
echo time (ms)	223
slice thickness (mm)	1
spacing	-0.6
matrix	288 x 288
field of view (cm)	23 x 20
3D FLAIR	
Plane	axial
Repetition time (ms)	4800
echo time (ms)	285
slice thickness (mm)	1.1
spacing	-1
matrix	240 x 240
field of view (cm)	23 x 22
3D T1-weighted TSE with contrast	Gadovist® 0.1 mmol/kg, administered before the 3D T2-weighted sequence

Supplementary table S4. Protocol details to acquire FBB PET data according with international guidelines [1]

DEVICE	*Siemens Biograph Vision 600*
Radiotracer	^{18}F-florbetaben (FBB)
Dose	300 MBq
EARLY FBB-PET/TC: 0-10′ p.i.	
acquisition	LIST MODE (DINAMIC)
reconstruction: 0-5′ and 0-10′ p.i.	
Matrix	440
Slice Thickness	1 mm
Corrections	Scatter, CT attenuation, Slice Coincidence
Zoom	2
Reconstruction Parameters	Iterativa (8 it; 5 sub)<br>Filter All-pass<br>TueX+TOF (ultra HD-PET)
STANDARD FBB-PET/TC: 90′ p.i.	
acquisition	LIST MODE (DINAMIC)
time	18 ‘
reconstruction:	
Matrix	440
Slice Thickness	1 mm
Corrections	Scatter, CT attenuation, Slice Coincidence
Zoom	2
Reconstruction Parameters	Iterativa (10 it; 5 sub)<br>Filter All-pass<br>TueX+TOF (ultra HD-PET)
Computed tomography	
Protocol (low dose)	35 mA / 120 kev
Reconstrution	HR 38, Sinogram Affirmed Iterative Rec (SAFIRE)
Slice Thickness	1
Intensity	3

1. Minoshima S, Drzezga AE, Barthel H, Bohnen N, Djekidel M, Lewis DH, Mathis CA, McConathy J, Nordberg A, Sabri O, Seibyl JP, Stokes MK, Van Laere K. SNMMI Procedure Standard/EANM Practice Guideline for Amyloid PET Imaging of the Brain 1.0. J Nucl Med. 2016 Aug;57(8):1316-22. doi: 10.2967/jnumed.116.174615. PMID: 27481605.

Supplementary table S5. Demographic characteristics, clinical assessment, and neuropsychological testing of the study cohort

**Characteristic (n= 20)**	**mean (SD) or n (%)**
MS onset age (y))	35.05 (10.72)
Sex	
Male	5 (25)
Female	15 (75)
Smoking habits	
Non-smoker	12 (60)
Former smoker	2 (10)
Current smoker	6 (30)
Education level	
Secondary education	10 (50)
Post-secondary non-tertiary education	3 (15)
University education	7 (35)
Employment status	
Employed	16 (80)
Student / Unemployed	4 (20)
Comorbidities (Charlson index)	
0	18 (90)
≥ 1	2 (10)
Initial clinical presentation	
Optic neuritis	6 (30)
Myelitis	7 (35)
Hemispheric syndrome	2 (10)
Brainstem syndrome	5 (25)
Nº of relapses	1.95 (1.15)
EDSS score	1.90 (1.09)
Progression disease scales	
T2FWT (seconds)	5.62 (1.19)
9HPT-D (seconds)	23.66 (5.06)
9HPT-ND (seconds)	24.07 (3.49)
MFIS-5 score	8.95 (6.37)
Cognitive functions	
SDMT (z-score)	-1.13 (0.96)
CVLT-II (z-score)	-1.35 (1.18)
BVMT (z-score)	-0.68 (1.51)
Stroop (z-score)	-0.55 (1.00)
Digit direct (z-score)	-0.53 (1.00)
Digit invers (z-score)	-0.91 (0.79)
SVF (z-score)	-0.50 (1.13)
PVF (z-score)	-0.85 (1.27)
Beck (points)	16.55 (12.97)
Quality of life	
EQ-5D (points)	68.75 (22.35)
High-disease-activity patients	
No	8 (40)
Sí	12 (60)

SD: standard deviation; EDSS: Expanded Disability Status Scale; T25FW : Timed 25-foot Walk, 9HPT-D: 9-hole Peg dominant side, 9HPT-ND: 9-hole Peg non-dominant side Test, MFIS: Modified Fatigue Impact Scale; SDMT: Symbol Digit Modalities Test; CVLT-II: California Learning Verbal Test – Second Edition; BVMT-R: Brief Visuospatial Memory Test-Revised; PVF: phonemic verbal fluency; SVF: semantic verbal fluency; EQ-5D: index and visual analogy scale.

Supplementary table S6. Structural (MRI) and functional (amyloid PET) neuroimaging characteristics of the study cohort.

**Characteristic** (n= 20)	**mean (SD) or n (%)**
DWM volume (cm^3)	13.94 (14.98)
NAWM volume (cm^3)	540.98 (38.59)
GM volume (cm^3)	968.63 (72.70)
White matter lesion burden	
(ml)	2 (10)
Low	8 (40)
High and non-confluent	10 (50)
High and confluent	
Other central nervous system involvement	
Spinal cord	11(55)
Brainstem	12 (60)
Cerebellum	9 (45)
Early FBB PET 0-5 minutes	
SUVmax in DWM	5.08 (1.36)
SUVmean in DWM	1.44 (0.51)
SUVmin in DWM	0.44 (0.19)
SUVmax in NAWM	7.20 (1.71)
SUVmean in NAWM	2.49 (0.68)
SUVmin in NAWM	0.33 (0.18)
SUVmax in cerebellum	5.58 (1.17)
SUVmean in cerebellum	2.89 (0.60)
SUVmin in cerebellum	0.10 (0.04)
SUVRmax in DWM	0.90 (0.11)
SUVRmean in DWM	0.49 (0.11)
SUVRmin in DWM	4.86 (2.31)
SUVRmax in NAWM	1.29 (0.11)
SUVRmean in NAWM	0.86 (0.14)
SUVRmin in NAWM	3.74 (2.17)
% of change SUVmax	-29.40 (8.56)
% of change SUVmean	-41.82 (13.44)
% of change SUVmin	46.52 (56.58)
Early FBB PET 0-10 minutes	
SUVmax in DWM	6.43 (2.29)
SUVmean in DWM	2.11 (0.79)
SUVmin in DWM	0.68 (0.36)
SUVmax in NAWM	9.57 (3.37)
SUVmean in NAWM	4.17 (1.98)
SUVmin in NAWM	0.43 (0.30)
SUVmax in cerebellum	7.02 (2.41)
SUVmean in cerebellum	3.74 (1.21)

SUVmin in cerebellum	0.13 (0.06)
SUVRmax in DWM	0.91 (0.10)
SUVRmean in DWM	0.56 (0.10)
SUVRmin in DWM	5.61 (3.20)
SUVRmax in NAWM	1.36 (0.11)
SUVRmean in NAWM	1.10 (0.42)
SUVRmin in NAWM	3.44 (2.32)
% of change SUVmax	-32.67 (7.80)
% of change SUVmean	-16.16 (109.47)
% of change SUVmin	82.76 (87.49)
Standard FBB PET	
SUVmax in DWM	3.31 (1.17)
SUVmean in DWM	1.82 (0.56)
SUVmin in DWM	0.70 (0.33)
SUVmax in NAWM	4.18 (1.34)
SUVmean in NAWM	2.21 (0.70)
SUVmin in NAWM	0.35 (0.24)
SUVmax in cerebellum	4.01 (1.41)
SUVmean in cerebellum	1.20 (0.38)
SUVmin in cerebellum	0.10 (0.05)
SUVRmax in DWM	0.84 (0.14)
SUVRmean in DWM	1.53 (0.16)
SUVRmin in DWM	8.25 (4.75)
SUVRmax in NAWM	1.06 (0.15)
SUVRmean in NAWM	1.85 (0.17)
SUVRmin in NAWM	3.72 (1.96)
% of change SUVmax	-20.98 (9.08)
% of change SUVmean	-16.93 (8.19)
% of change SUVmin	136.15 (120.89)

SD: standard deviation; DWM: damage white matter; NAWM: normally-appearing white matter; GM: grey matter, SUVmax: Maximum Standardized Uptake Value, SUVmean: Mean Standardized Uptake Valu, SUVmin: Minimum Standardized Uptake Value; SUVR; SUV relative to cerebellum.

Supplementary table S7. Comparative analysis of Standardized Uptake Value (SUV) in damage white matter (DMG) vs. normally-appearing white matter (NAWM) in the early (eFBB) y standard (sFBB) phases of [18F] Florbetaben (FBB) PET.

	**eFBB PET (0-5´p.i.)**			**eFBB PET (0-10´p.i.)**			**sFBB PET (90´p.i.)**		
	DWM mean (SD)	NAWM mean (SD)	p	DWM mean (SD)	NAWM mean (SD)	p	DWM mean (SD)	NAWM mean (SD)	p
**SUVmax**	5.08 (1.36)	7.20(1.71)	<0.001	6.43 (2.29)	9.57 (3.37)	<0.001	3.31 (1.17)	4.17 (1.34)	<0.001
**SUVmean**	1.44 (0.41)	2.49 (0.68)	<0.001	2.11 (0.79)	4.17 (1.98)	<0.001	1.82 (0.56)	2.21 (0.70)	<0.001
**SUVmin**	0.44 (0.19)	0.33 (0.18)	<0.001	0.68 (0.36)	0.43 (0.31)	<0.001	0.70 (0.33)	0.35 (0.24)	<0.001
**SUVRmax**	0.90 (0.11)	1.29 (0.11)	<0.001	0.91 (0.96)	1.36 (0.11)	<0.001	0.84 (0.14)	1.06 (0.15)	<0.001
**SUVRmean**	0.49 (0.11)	0.86 (0.14)	<0.001	0.56 (0.10)	1.10 (0.42)	<0.001	1.53 (0.16)	1.85 (0.17)	<0.001
**SUVRmin**	4.86 (2.31)	3.71 (2.17)	<0.001	5.61 (3.20)	3.44 (2.32)	<0.001	8.25 (4.75)	3.72 (1.96)	<0.001

SD: standard deviation; DWM: damage white matter; NAWM: normally-appearing white matter; SUVmax: Maximum Standardized Uptake Value, SUVmean: Mean Standardized Uptake Valu, SUVmin: Minimum Standardized Uptake Value; SUVR; SUV relative to cerebellum.

Supplementary table S8. Comparative analysis of Standardized Uptake Value ratios (SUVR) between the early (eFBB) and standard (sFBB) phases of [18F]Florbetaben PET in both damage white matter (DWM) and normal-appearing white matter (NAWM).

**Variable**		**eFBB PET (0-5´p.i.) mean (SD)**	**eFBB PET (0-10´p.i.) mean (SD)**	**sFBB PET (90´p.i.) mean (SD)**	**p**
**DWM**	SUVRmax	0.90 (0.11)	0.91 (0.96)	0.84 (0.14)	0.094
	SUVRmean	0.49 (0.10)	0.56 (0.97)	1.53 (0.16)	<0.001
	SUVRmin	4.63 (2.21)	5.61 (3.20)	8.25 (4.75)	<0.001
**NAWM**	SUVRmax	1.28 (0.11)	1.36 (0.11)	1.06 (0.15)	<0.001
	SUVRmean	0.86 (0.14)	1.10 (0.42)	1.85 (0.87)	<0.001
	SUVRmin	3.74 (2.17)	3.44 (2.32)	3.72 (1.96)	0.739
**% of change SUV**	SUVmax	-29.40 (8.56)	-32.67 (7.80)	-20.98 (9.08)	<0.001
	SUVmean	-41.82 (13.44)	-16.16 (109.47)	-16.93 (8.19)	<0.001
	SUVmin	46.52 (56.58)	82.76 (87.49)	136.15 (120.89)	0.015

SD: standard deviation; DWM: damage white matter; NAWM: normally-appearing white matter; SUVmax: Maximum Standardized Uptake Value, SUVmean: Mean Standardized Uptake Valu, SUVmin: Minimum Standardized Uptake Value; SUVR; SUV relative to cerebellum.

Supplementary figure S1. Multiple Line Chart showing comparative analysis of Standardized Uptake Value ratios (SUVR) between the early (eFBB) and standard (sFBB) phases of [18F]Florbetaben PET in both damage white matter (DWM) and normal-appearing white matter (NAWM).

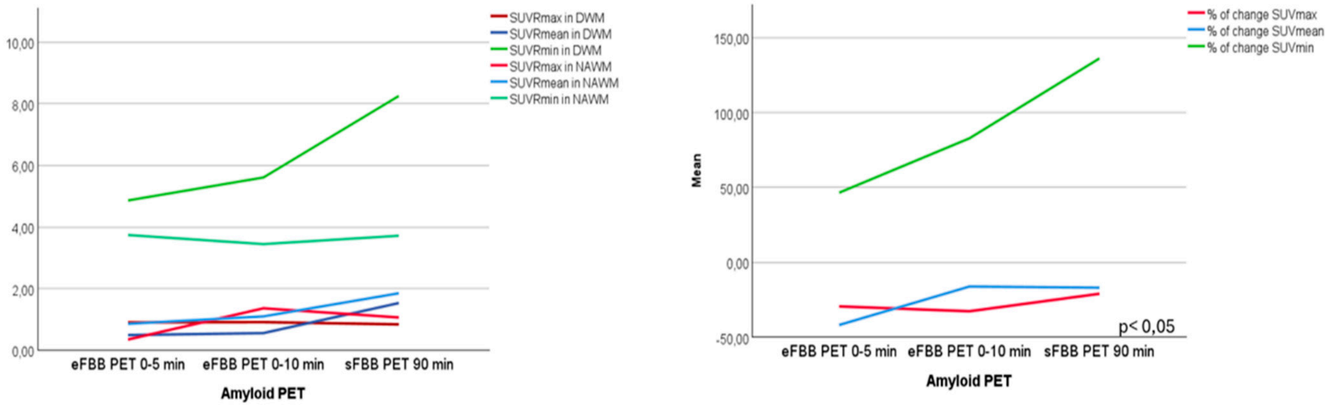

Supplementary figure S2. Scatter plot showing the correlation between EDSS score and the percentage of change in SUVmax calculated from standard-phase [^{18}F]Florbetaben (sFBB) PET

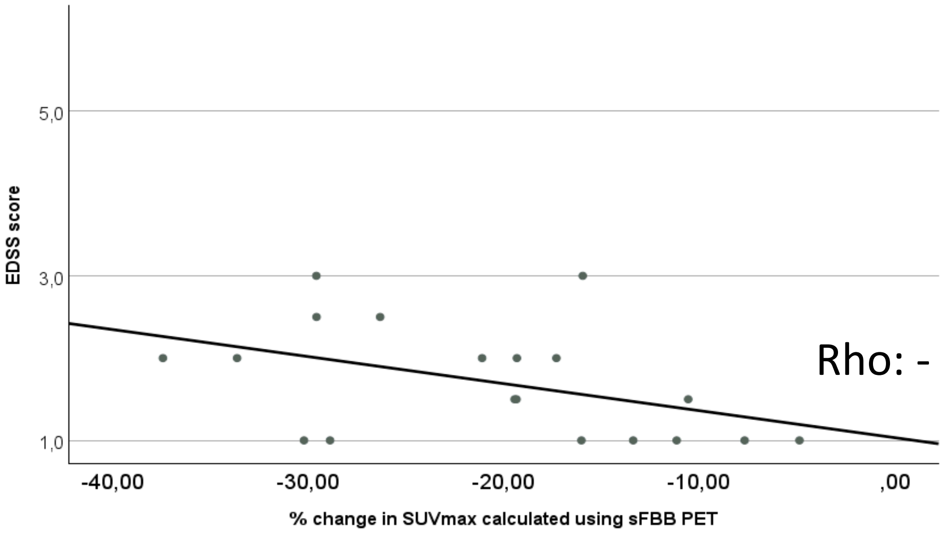

Supplementary table S9. Correlation between quantitative parameters from the early (eFBB) and standard (sFBB) phases of [18F]Florbetaben PET and clinical variables related to disease activity and progression.

Variable		Nº relapses	EDSS	T25FW	9HPTD	9HPTND	SDMT	CVLT	BVMT	BDP-II	MFIS-5	EQ5D	Stroop	Digit direct	Digit inverse	FVS	FVF
**eFBB PET (0-5´p.i.)**	SUVRmax in DWM	**-0,504***	**-0,501***	0,002	-0,357	-0,146	0,009	0,217	-0,035	-0,282	**-0,450***	0,268	0,162	-0,251	0,019	0,055	0,078
	SUVRmean in DWM	**-0,541***	-0,383	-0,247	-0,29	-0,296	-0,106	-0,141	-0,286	-0,266	**-0,565***	0,054	0,295	-0,443	0,016	-0,155	0,087
	SUVRmin in DWM	-0,257	0,371	0,168	0,275	0,302	**-0,516***	**-0,564***	-0,422	**0,570***	0,316	**-0,468***	-0,207	-0,233	0,231	-0,201	-0,186
	SUVRmax in NAWM	-0,066	-0,083	0,314	-0,029	0,078	0,048	0,192	-0,119	-0,08	-0,199	-0.147	-0,18	-0,197	-0,176	-0,19	-0,154
	SUVRmean in NAWM	**-0,478***	0,152	0,214	-0,051	0,078	-0,16	0,113	-0,146	0,255	-0,031	-0.081	-0,055	-0,109	0,267	0,012	-0,089
	SUVRmin in NAWM	-0,095	0,357	-0,087	0,27	0,096	-0,376	**-0,649***	-0,36	0,343	0,252	-0.331	-0,057	-0,105	0,21	-0,076	-0,092
	% of change SUVmax	-0,399	-0,31	-0,229	-0,282	-0,137	-0,156	-0,098	-0,089	-0,099	-0,303	0.065	0,385	-0,291	0,099	0,109	0,179
	% of change SUVmean	-0,071	-0,429	-0,393	-0,224	-0,361	-0,023	-0,286	-0,193	**-0,542***	**-0,579****	0.092	0,144	-0,336	-0,345	-0,258	0,063
	% of change SUVmin	-0,32	0,033	0,204	**-0,483***	0,167	0,266	**0,477***	0,239	-0,007	-0,079	-0.095	-0,063	-0,032	-0,005	-0,077	0,048
**eFBB PET (0-10´p.i.)**	SUVRmax in DWM	-0,387	**-0,513***	-0,118	**-0,623****	-0,232	0,225	**0,503***	0,221	-0,365	**-0,450***	**0,499***	0,39	-0,239	-0,094	0,327	0,293
	SUVRmean in DWM	**-0,596***	-0,191	-0,238	-0,219	-0,032	-0,137	-0,143	-0,218	0,017	-0,335	-0.207	0,336	-0,319	0,231	-0,024	0,133
	SUVRmin in DWM	-0,282	0,376	0,038	0,242	0,271	**-0,539***	**-0,583***	-0,383	**0,444***	0,229	**-0,490***	-0,228	-0,315	0,038	-0,205	-0,116
	SUVRmax in NAWM	0,06	-0,117	**0,540***	0,011	0,154	0,088	0,299	-0,025	-0,149	-0,063	0.005	-0,248	-0,153	-0,179	-0,303	-0,186
	SUVRmean in NAWM	**-0,736****	-0,054	0,146	-0,1	0,041	0,038	0,33	0,093	0,308	-0,047	-0.071	0,04	0,019	0,417	0,105	0,077
	SUVRmin in NAWM	0,06	0,369	-0,303	0,123	-0,078	-0,323	**-0,592***	-0,324	0,044	0,103	-0.167	0,022	-0,175	0,03	0,02	0,006
	% of change SUVmax	-0,221	-0,184	-0,387	**-0,472***	-0,149	0,029	0,059	0,078	-0,075	-0,171	0.295	**0,498**	-0,199	0,029	**0,488***	0,33
	% of change SUVmean	0,397	-0,119	-0,216	-0,074	-0,03	-0,11	-0,386	-0,203	-0,345	-0,237	-0.113	0,026	-0,193	-0,361	-0,232	-0,107
	% of change SUVmin	**-0,485***	0,192	**0,453***	-0,026	**0,445***	-0,12	0,168	0,074	**0,452**	0,224	-0.426	-0,305	-0,077	0,178	-0,225	-0,105
**sFBB PET (90´p.i.)**	SUVRmax in DWM	-0,173	-0,13	0,158	-0,241	0,143	-0,164	0,066	-0,232	-0,032	-0,246	-0.185	-0,008	**-0,578***	-0,197	-0,081	-0,077
	SUVRmean in DWM	-0,057	0,134	0,203	-0,215	0,132	0,026	-0,038	-0,063	0,12	-0,008	-0.028	-0,046	0,02	0,096	0,05	0,003
	SUVRmin in DWM	-0,226	0,159	0,071	0,106	-0,13	-0,027	-0,232	-0,145	0,199	0,055	-0.212	-0,041	-0,055	0,305	-0,181	0,034
	SUVRmax in NAWM	-0,274	0,008	0,15	0,072	0,286	-0,296	0,124	-0,037	0,23	-0,034	-0.260	0,019	**-0,484***	-0,17	0,11	0,129
	SUVRmean in NAWM	0,276	0,196	0,256	0,392	0,414	-0,369	-0,303	-0,196	0,278	0,243	-0.195	-0,208	0,107	0,071	-0,038	-0,14
	SUVRmin in NAWM	0,047	0,023	-0,211	0,044	-0,336	-0,011	-0,195	-0,095	-0,029	-0,062	-0.052	0,036	-0,094	-0,079	-0,128	0,021
	% of change SUVmax	-0,183	**-0,446***	-0,026	**-0,477***	-0,1	0,129	0,045	-0,25	-0,218	-0,36	0.208	0,185	-0,267	-0,018	-0,045	-0,051
	% of change SUVmean	**-0,454***	-0,105	-0,061	**-0,613****	-0,22	0,337	0,317	0,233	-0,154	-0,332	0.066	0,163	-0,077	0,085	0,073	0,194
	% of change SUVmin	-0,206	0,334	**0,440***	-0,035	0,259	-0,098	-0,15	-0,194	0,303	0,19	-0.220	-0,222	-0,002	0,347	-0,153	-0,18

*Correlation is significant at the 0.05 level (two-tailed).

**Correlation is significant at the 0.01 level (two-tailed).

Red tones indicate positive correlations (values ranging from 0 to +1), blue tones indicate negative correlations (values ranging from 0 to –1), and white indicates no correlation.
